# A Multiple QTL-Seq Strategy Delineates Potential Genomic Loci Governing Flowering Time in Chickpea

**DOI:** 10.3389/fpls.2017.01105

**Published:** 2017-07-11

**Authors:** Rishi Srivastava, Hari D. Upadhyaya, Rajendra Kumar, Anurag Daware, Udita Basu, Philanim W. Shimray, Shailesh Tripathi, Chellapilla Bharadwaj, Akhilesh K. Tyagi, Swarup K. Parida

**Affiliations:** ^1^National Institute of Plant Genome Research New Delhi, India; ^2^International Crops Research Institute for the Semi-Arid Tropics Patancheru, India; ^3^U.P. Council of Agricultural Research Lucknow, India; ^4^Division of Genetics, Indian Agricultural Research Institute New Delhi, India

**Keywords:** chickpea, flowering time, multiple QTL-seq, QTL, SNP

## Abstract

Identification of functionally relevant potential genomic loci using an economical, simpler and user-friendly genomics-assisted breeding strategy is vital for rapid genetic dissection of complex flowering time quantitative trait in chickpea. A high-throughput multiple QTL-seq strategy was employed in two inter (*Cicer arietinum desi* accession ICC 4958 × *C reticulatum* wild accession ICC 17160)- and intra (ICC 4958 × *C. arietinum kabuli* accession ICC 8261)-specific RIL mapping populations to identify the major QTL genomic regions governing flowering time in chickpea. The whole genome resequencing discovered 1635117 and 592486 SNPs exhibiting differentiation between early- and late-flowering mapping parents and bulks, constituted by pooling the homozygous individuals of extreme flowering time phenotypic trait from each of two aforesaid RIL populations. The multiple QTL-seq analysis using these mined SNPs in two RIL mapping populations narrowed-down two longer (907.1 kb and 1.99 Mb) major flowering time QTL genomic regions into the high-resolution shorter (757.7 kb and 1.39 Mb) QTL intervals on chickpea chromosome 4. This essentially identified regulatory as well as coding (non-synonymous/synonymous) novel SNP allelic variants from two *efl1* (early flowering 1) and *GI* (*GIGANTEA*) genes regulating flowering time in chickpea. Interestingly, strong natural allelic diversity reduction (88–91%) of two known flowering genes especially mapped at major QTL intervals as compared to that of background genomic regions (where no flowering time QTLs were mapped; 61.8%) in cultivated vis-à-vis wild *Cicer* gene pools was evident inferring the significant impact of evolutionary bottlenecks on these loci during chickpea domestication. Higher association potential of coding non-synonymous and regulatory SNP alleles mined from *efl1* (36–49%) and *GI* (33–42%) flowering genes for early and late flowering time differentiation among chickpea accessions was evident. The robustness and validity of two functional allelic variants-containing genes localized at major flowering time QTLs was apparent by their identification from multiple intra-/inter-specific mapping populations of chickpea. The functionally relevant molecular tags delineated can be of immense use for deciphering the natural allelic diversity-based domestication pattern of flowering time and expediting genomics-aided crop improvement to develop early flowering cultivars of chickpea.

## Introduction

Chickpea (*Cicer arietinum* L.) is one of the vital food legume crops represented by two of its major cultivar types- *desi* and *kabuli* (Kumar et al., 2011) which are thought to be domesticated along with the wild ancestor *C. reticulatum* at Fertile Crescent around 10000 years ago (Burger et al., 2008; Toker, 2009). Development of early-flowering/maturing stress tolerant cultivars with high seed and pod yield is the prime objective of the present genomics-assisted breeding research in chickpea (Hegde, 2010; Zaman-Allah et al., 2011). The number of days to flowering is a major seed and pod-yield component trait that highly acclimatizes with climate change, diverse environmental and a/biotic stress factors and photoperiod response along with various other growth/developmental-related traits (Aryamanesh et al., 2010; Kashiwagi et al., 2013; Daba et al., 2016). Therefore, implication of flowering time in defining productivity as well as developing stress tolerant cultivars is well-documented in chickpea. Collectively, this infers that flowering time is a complex quantitative trait and it is governed by multiple major as well as minor genes/QTLs (quantitative trait loci). A strong impact of a known major evolutionary bottleneck- vernalization- on flowering time response during chickpea domestication infers that the flowering time is a most important domestication trait selected during breeding of presently cultivated *desi* and *kabuli* accessions (Abbo et al., 2014). The genetic dissection of complex flowering time quantitative trait by identifying the functionally relevant potential genes/alleles colocalized at QTLs governing this major yield component and domestication trait is thus imperative for their broader effective practical applicability in marker-aided genetic improvement of chickpea.

Significant progress has been made to decipher the complex genetic inheritance characteristics and molecular genetic dissection of flowering time trait in chickpea (Anbessa et al., 2006; Cobos et al., 2007; Pierre et al., 2008, 2011; Aryamanesh et al., 2010; Zhang et al., 2013). This involves identification of four different major early flowering (*efl*) gene loci/allelic variants [*efl1, efl2*/*ppd* (photoperiod), *efl3* and *efl4*] controlling varied flowering time trait adaptation characteristics in multiple chickpea accessions (Hegde, 2010; Gaur et al., 2014; Weller and Martínez, 2015). Additionally, this includes colocalization of various known flowering time gene homologs [like *Efl1, Efl2, LFY* (LEAFY) and FT (flowering time) gene families] within the low-resolution major flowering time QTL regions mapped on chickpea chromosomes (Cho et al., 2002; Anbessa et al., 2006; Lichtenzveig et al., 2006; Cobos et al., 2007, 2009; Aryamanesh et al., 2010; Hossain et al., 2010; Rehman et al., 2011; Vadez et al., 2012; Jamalabadi et al., 2013; Varshney et al., 2014).

Substantial efforts have also been made to understand the complex gene regulatory networks and transcriptional modules governing flower development in a *desi* chickpea accession (ICC 4958) through NGS (next-generation sequencing)-based global transcriptome sequencing strategy (Singh et al., 2013). The deployment of these differentially expressed candidate gene-derived SNPs in association mapping and their subsequent integration with GWAS (genome-wide association study), high-resolution QTL mapping, differential transcript profiling, molecular haplotyping have delineated tissue/stage (flower bud/flower)-specific differentially regulated potential candidate genes underlying major QTLs regulating flowering time at a whole genome level in chickpea (Das et al., 2015b; Upadhyaya et al., 2015). Until yet, none of these identified genes harboring major flowering time QTLs have been validated in multiple genetic backgrounds (mapping populations) and identified through map-based cloning that could be employed for marker-aided genetic enhancement of chickpea. This could be restrained due to low marker genetic polymorphism particularly between parents of multiple intra-/inter-specific mapping populations along with limited accessibility of large size mapping populations and high-density genetic linkage maps of chickpea. An alternative genome-wide approach is thus essential for quick identification and molecular mapping (fine-mapping/map-based isolation) of high-resolution flowering time QTLs/genes in order to drive genomics-led crop improvement in chickpea.

For genetic mapping of major flowering time QTLs, conventional QTL mapping approach that primarily involves genotyping of large-scale SSR (simple sequence repeat) and SNP (single nucleotide polymorphism) markers among mapping individuals of diverse inter-/intra-specific populations is found much expedient in chickpea (Anbessa et al., 2006; Lichtenzveig et al., 2006; Cobos et al., 2007, 2009; Aryamanesh et al., 2010; Hossain et al., 2010; Gowda et al., 2011; Rehman et al., 2011; Vadez et al., 2012; Jamalabadi et al., 2013; Stephens et al., 2014; Varshney et al., 2014). This approach essentially identified a diverse array of low-resolution longer marker confidence interval spanning major QTLs associated with chickpea flowering and maturation time (Cho et al., 2002; Anbessa et al., 2006; Lichtenzveig et al., 2006; Cobos et al., 2007, 2009; Aryamanesh et al., 2010; Hossain et al., 2010; Rehman et al., 2011; Vadez et al., 2012; Jamalabadi et al., 2013; Varshney et al., 2014).

The freely accessible draft genome sequences are found much proficient to accelerate genome and transcriptome resequencing of diverse *desi, kabuli* and wild accessions that are most commonly utilized as parents for generating diverse intra- and inter-specific mapping populations of chickpea (Jain et al., 2013; Varshney et al., 2013; Parween et al., 2015). Aside genomic resources, multiple genetic resources including advanced generation recombinant inbred lines (RILs) and back-cross mapping populations as well as core/mini-core germplasm accessions exhibiting a broader range of phenotypic variation for flowering time trait are now available in chickpea (Upadhyaya et al., 2001, 2008; Gaur et al., 2014). All these available genetic and genomic resources essentially have assisted in utilization of a high-throughput NGS-based QTL-seq strategy vis-à-vis a commonly adopted traditional QTL mapping approach for fast genome-wide scanning and genetic mapping of major QTLs controlling various quantitative agronomic traits (for instance, 100-seed weight, pod number and root/total plant dry weight ratio) in chickpea (Das et al., 2015a, 2016; Singh et al., 2016).

To complement this, a multiple QTL-seq assay that relies on QTL-seq analysis in multiple mapping populations generated by inter-crossing of common parental accessions, has been developed currently as a most promising genome-wide strategy for QTL mapping at a high-resolution scale (Das et al., 2016). Essentially, multiple QTL-seq involves whole genome NGS resequencing of DNA bulks (exhibiting two utmost contrasting phenotypic traits) constituted from homozygous individuals of multiple mapping populations comprising at least single common parent. This approach is found most promising based on its potential to validate QTL-seq-derived major QTLs identified from individual preliminary as well as advanced generation intra-/inter-specific mapping population in multiple mapping populations of diverse genetic backgrounds. Moreover, utility of this approach is clearly evident from its efficacy to narrow-down each QTL-seq originated sizeable long QTL genomic intervals into functionally relevant potential candidate genes governing important agronomic traits (for instance, pod number) in chickpea (Das et al., 2016).

Considering usefulness and broader practical applicability, multiple QTL-seq assay can be employed for rapid genome-wide scanning and fine-mapping (positional cloning) of trait-linked major genes and natural allelic-variants colocalized at robust QTLs (well-validated in multiple mapping populations) in chickpea with minimal resource expenses. This will collectively enrich our understanding on complex genetic architecture and evolutionary pattern influencing flowering time quantitative trait variation during domestication of chickpea in order to expedite its genomics-assisted crop improvement. In view of afore-mentioned possibilities, a multiple QTL-seq strategy was employed in two inter- and intra-specific RIL (recombinant inbred lines) mapping populations- (*C. arietinum desi* accession ICC 4958 × *C. reticulatum* wild accession ICC 17163) and (ICC 4958 × *C. arietinum kabuli* accession ICC 8261)- at a genome-wide scale to delineate major genomic (gene) regions and novel natural allelic variants underlying the QTLs associated with flowering time in chickpea.

## Materials and Methods

### Development and Phenotyping of RIL Mapping Populations for Flowering Time

Two inter- and intra-specific F_9_ RIL mapping populations- (ICC 4958 × ICC 17163, population size: 260) and (ICC 4958 × ICC 8261, 204)- with contrasting flowering time trait were developed by single seed descent method. As per field phenotyping at International Crops Research Institute for the Semi-Arid Tropics (ICRISAT), ICC 4958 (traditional cultivar/landrace, originated from India) is an early flowering chickpea accession with DTF (days to 50% flowering time) of 43 days. In contrast, ICC 17163 (wild accession) and ICC 8261 (traditional cultivar/landrace) originated from Turkey are late flowering chickpea accessions with DTF of 85 and 61 days, respectively. The *desi* chickpea accession ICC 4958 was considered as a common parent for both mapping populations generated.

For phenotyping, the mapping individuals and parents of both RIL populations were grown and phenotyped in the field as per RCBD (randomized complete block design) with two replications at two diverse eco-geographical regions [(ICRISAT, Patancheru, Hyderabad: latitude 17° 3′ N/longitude 77° 2′ E from October to February) and (National Institute of Plant Genome Research (NIPGR), New Delhi: 28° 4′ N/77° 2′ E from November to March)] of India for two successive years (2013 and 2014) during crop growing season. In addition, these parents and RIL individuals were grown in the greenhouse to determine the flowering time response of these mapping individuals under both long- and short-day conditions at 22 ± 2^0^C following Upadhyaya et al. (2015). Ten to fifteen representative plants were screened from each mapping individual and parental accession of two RIL populations, and DTF of each individual/accession was calculated as per Upadhyaya et al. (2015). The homogeneity of RIL mapping populations across two locations/years as well as major parameters contributing to genetic inheritance characteristics such as frequency distribution, CV (coefficient of variation), H^2^ (broad-sense heritability) of DTF trait among RIL individuals were determined as per Bajaj et al. (2015b). To evaluate the genetic inheritance pattern of flowering time trait, the interactions of mapping individuals/parents (G) with their phenotyping environments (E; like years and locations) were calculated using ANOVA (analysis of variance).

### Whole Genome Resequencing and Multiple QTL-Seq Analysis

We selected 10 of each early and late flowering homozygous mapping individuals belonging to two extreme ends of DTF normal frequency distribution curve from each of the two RIL populations of ICC 4958 × ICC 17163 and ICC 4958 × ICC 8261 for QTL-seq study. Prior to inclusion of these selected 20 RIL mapping individuals in QTL-seq analysis, the homozygous genetic constitution of these individuals from both RIL populations for either of the early and late flowering trait was assured using their DTF field phenotyping data and genome-wide SSR markers-based genotyping information as per Das et al. (2015a, 2016).

The genomic DNA was isolated from constituted DNA bulks- early days to 50% flowering time bulk (EDTFB) and late days to 50% flowering time bulk (LDTFB) as well as parents of mapping populations using QIAGEN DNeasy kit (QIAGEN, United States) following manufacturer’s instructions. The quantity and quality of isolated genomic DNA was ensured by Qubit 2.0 Fluorometer (Invitrogen Life Technologies, United States) and Bioanalyzer 2100 (Agilent Technologies, United States), respectively. About 1 μg of high-quality genomic DNA of each sample was utilized for library preparation using Illumina TruSeq DNA PCR-Free Library Preparation Kit according to the manufacturer’s protocol. The libraries were processed for paired-end sequencing (100-nucleotide long reads) using Illumina HiSeq2000 platform (Illumina Technologies, United States) and raw sequence data were filtered through standard Illumina pipeline. The FASTQ sequences were further processed through NGS QC Toolkit v2.3 (Patel and Jain, 2012) to remove low-quality including primer/adaptor contaminated sequence reads. The filtered reads with a minimum phred Q-score of 30 across > 95% of nucleotide sequence were considered as high-quality.

Recently, a whole genome high-quality sequence assembly, including large size (510.9 Mb) chromosome pseudomolecule (334 Mb) and scaffolds of *desi* (ICC 4958) chickpea genome are freely available in public domain (Parween et al., 2015). Accordingly, a *desi* chickpea accession ICC 4958 for which the genome sequence is available was utilized as one of the common parent in two RIL mapping populations developed for our QTL-seq analysis. Therefore, we preferably utilized the latest released *desi* reference genome sequence as an anchor to mine resequencing-based SNPs from mapping parents and bulks for QTL-seq study at a whole genome level in chickpea.

High-quality sequence reads generated from parental accessions and bulks (EDTFB and LDTFB) were mapped onto reference *desi* chickpea genome using BWA with default parameters (Parween et al., 2015). Consequently, the uniquely mapped sequence reads were normalized in accordance with read coverage-depth among mapping parents and RIL individuals forming EDTFB and LDTFB bulks (Supplementary Table S1). The mined homozygous high-quality SNPs (minimum sequencing read-depth 10 with mean base quality ≥ 20) exhibiting differentiation between parents as well as between EDTFB and LDTFB were structurally and functionally annotated with respect to reference *desi* chickpea genome following Kujur et al. (2015a,b,c). As per the earlier defined recommended parameters of Takagi et al. (2013), Lu et al. (2014), and Das et al. (2015a, 2016), SNP-index and Δ (SNP-index)-led QTL-seq assay was employed in two RIL mapping populations individually to identify major DF QTLs in chickpea. The subtraction of SNP-index (percentage of SNPs-supporting sequence reads completely different from reference *desi* genome) between EDTFB and LDTFB was measured as Δ (SNP-index). Following the representation of genomic fragments obtained from ICC 4958 (early DTF) and ICC 17163/ICC 8261 (late DTF) in entire genome sequences, the SNP-index was calculated as “0” and “1,” respectively. The major genomic regions underlying QTL-seq derived DTF QTLs were ascertained by Δ (SNP-index) which is altogether different from 0 at a 99% significance level and thereby, considered to be highly significant QTLs governing flowering time in chickpea. A 10 Mb window-size and 1 kb increment sliding window approach was used to evaluate the mean distribution of Δ (SNP-index) of SNPs physically mapped across chromosomes in a target genomic interval. The SNP-index plots of EDTFB and LDTFB for two individual RIL mapping populations and null hypothesis statistical confidence intervals of Δ (SNP-index) were obtained to determine the accuracy and validity of QTL-seq derived QTLs following Takagi et al. (2013), Lu et al. (2014), and Das et al. (2015a, 2016).

### Natural Allelic Diversity in Flowering Genes

The novel natural SNP allelic variants of flowering time-associated candidate genes underlying major DTF QTLs validated by multiple QTL-seq assay, were genotyped using the genomic DNA of 172 including 93 cultivated (39 *desi* and 53 *kabuli* accessions) and 79 wild chickpea accessions [*C. reticulatum* (14 accessions), *C. echinospermum* (8), *C. judaicum* (22), *C. bijugum* (19) and *C. pinnatifidum* (15) and *C. microphyllum* (1); Bajaj et al. (2015a)] through Sequenom MALDI-TOF MassARRAY^1^ as per Saxena et al. (2014a,b). The SNP allelic genotyping data generated among chickpea accessions were analyzed with TASSEL v5.0 (100-kb non-overlapping sliding window) to estimate the various nucleotide diversity parameters (𝜃π and Tajima’s D) following Bajaj et al. (2015a) and Kujur et al. (2015a). For association analysis, the genotyping data of SNPs derived from flowering time-associated genes was integrated with DTF field and greenhouse-based phenotyping information, population structure ancestry coefficient (Q-matrix), kinship-matrix (K) and principal component analysis (PCA; P) data of 172 accessions following the detailed methods as described by Upadhyaya et al. (2015) to determine the SNP allele effect on early and late flowering time differentiation in chickpea.

## Results

### Genetic Inheritance Pattern of Flowering Time in Mapping Populations

**FIGURE 1:**
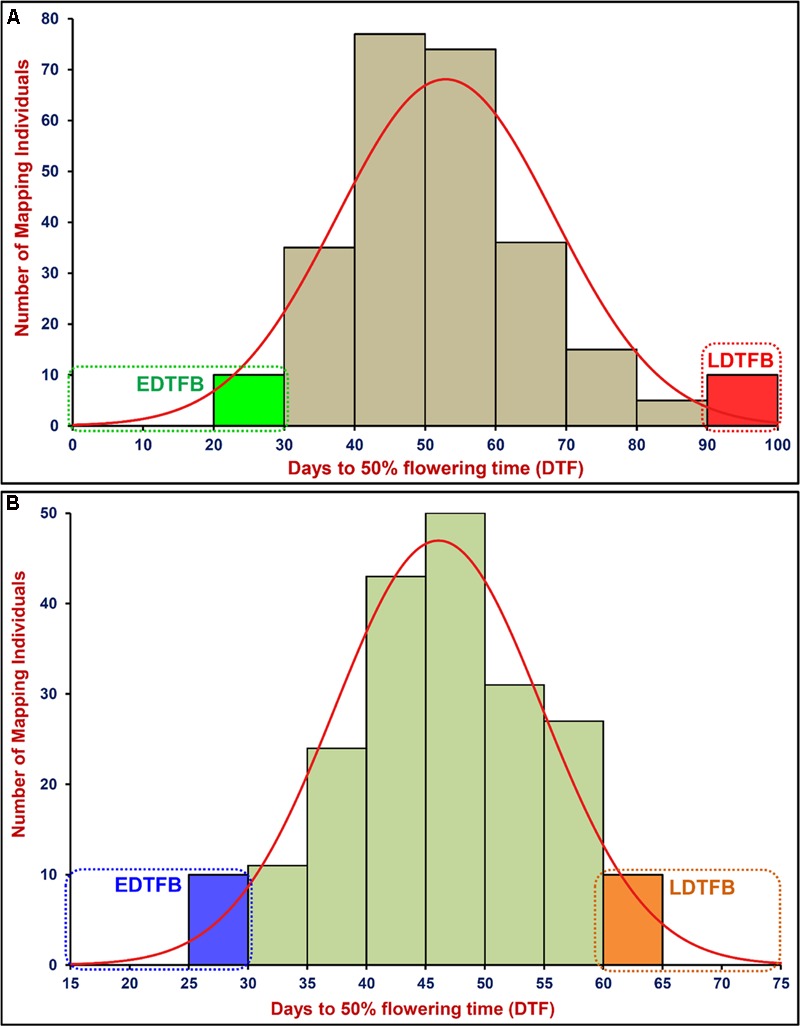
Frequency distribution of mean days to 50% flowering time (DTF) trait variation measured among 260 and 204 mapping individuals and parents of two F_9_ inter-/intra-specific RIL populations of ICC 4958 × ICC 17163 **(A)** and ICC 4958 × ICC 8261 **(B)**, respectively. These were grown and phenotyped in field at two different geographical locations and green house (long- and short-day photoperiod) for 2 years. EDTFB/LDTFB: early/late days to 50% flowering time bulk.

A significant difference of DTF trait was observed on phenotyping in field at two diverse geographical locations and in green house (long- and short-day photoperiod conditions) for 2 years. This varied from 25.8 to 99.5 days with 47.1 to 57.8 days mean ± 13.5–16.1 days standard deviation (SD) of DTF trait with 25.6–32.1% CV and 79–86% H^2^ among 260 individuals and parents of an inter-specific RIL mapping population [ICC 4958 (33.6–46.8 days mean ± 2.1–3.7 days SD) × ICC 17163 (85.1–91.5 days mean ± 3.3–4.1 days SD)] (**Table 1**). A wider phenotypic variation (varied from 25.7 to 70.9 days with 46.1 to 53.9 days mean ± 8.1–9.2 days SD) for DTF with 17.2–19.5% CV and 79–83% H^2^ was detected among 204 individuals and parents of an another intra-specific RIL mapping population [ICC 4958 (32.5–49.7 mean ± 2.7–3.6 SD) × ICC 8261 (57.9–67.5 mean ± 2.8–4.1 SD)] phenotyped similarly in field at two different geographical locations and in green house (long- and short-day photoperiod) for 2 years (**Table 1**). A significant (*P* < 0.0001) difference in DTF of individuals representing both RIL mapping populations grown under long- and short-day photoperiod conditions at green-house across 2 years was apparent. We observed a continuous variation-based normal frequency distribution along with a bi-directional transgressive segregation of DTF trait in these two RIL mapping populations (**Figures 1A,B**).

**Table 1:** Diverse statistical measures-based DTF (days to 50% flowering time) trait variation determined in two intra- and inter-specific chickpea RIL mapping populations grown in field at two diverse geographical locations of India and in greenhouse (long- and short-day conditions) for 2 years.

			Parental accessions		F_9_ RIL mapping individuals			
Mapping	Geographical		ICC 4958	ICC 17163	Mean ± SD;	Range	Coefficient of	Broad-sense
populations	locations	Years	(Mean ± SD; days)	(Mean ± SD; days)	(days)	(days)	variation (CV%)	Heritability (H^2^%)
ICC 4958 × ICC 17163 (260 F_9_ RILs)	Patancheru (Hyderabad)-field	2012	43.0 ± 2.5	85.4 ± 3.4	52.9 ± 15.3	27.1–97.2	28.9	85
		2013	43.2 ± 2.1	85.1 ± 3.5	52.7 ± 13.5	26.8–98.2	25.6	83
	New Delhi-field	2012	42.8 ± 2.6	85.5 ± 3.6	52.1 ± 14.7	26.1–96.3	28.2	86
		2013	43.1 ± 2.8	85.4 ± 3.9	53.4 ± 13.8	25.8–97.1	25.8	85
	Patancheru-green house (Long-day condition)	2012	47.5 ± 3.4	89.7 ± 4.1	56.1 ± 16.1	31.1–99.2	28.7	81
		2013	46.8 ± 3.7	90.2 ± 3.9	57.8 ± 15.5	30.6–99.5	26.8	80
	Patancheru-green house (Short-day condition)	2012	35.4 ± 3.7	90.7 ± 3.7	48.5 ± 15.4	22.4–89.7	31.8	79
		2013	33.6 ± 3.4	91.5 ± 3.3	47.1 ± 15.1	23.1–90.1	32.1	80

			**Parental accessions**		**F_9_ RIL mapping individuals**			
**Mapping**	**Geographical**		**ICC 4958**	**ICC 8261**	**Mean ± SD;**	**Range**	**Coefficient of**	**Broad-sense**
**populations**	**locations**	**Years**	**(Mean ± SD; days)**	**(Mean ± SD; days)**	**(days)**	**(days)**	**variation (CV%)**	**Heritability (H^2^%)**

ICC 4958 × ICC 8261 (204 F_9_ RILs)	Patancheru (Hyderabad)-field	2012	43.1 ± 2.7	61.0 ± 3.1	46.1 ± 8.7	25.7–64.9	18.9	83
		2013	43.5 ± 2.9	61.5 ± 3.4	46.5 ± 8.9	26.3–65.9	19.1	81
	New Delhi-field	2012	43.1 ± 3.1	60.8 ± 3.2	46.7 ± 9.1	26.5–65.1	19.5	80
		2013	42.8 ± 3.3	61.5 ± 2.8	46.9 ± 8.8	26.8–65.4	18.8	82
	Patancheru-green house (Long-day condition)	2012	48.2 ± 2.9	66.4 ± 4.1	49.5 ± 8.1	29.1–70.9	16.4	80
		2013	49.7 ± 3.4	67.5 ± 3.9	48.9 ± 8.4	28.9–69.2	17.2	80
	Patancheru-green house (Short-day condition)	2012	32.5 ± 3.6	58.7 ± 3.5	51.8 ± 9.2	30.5–69.5	17.8	80
		2013	34.2 ± 3.1	57.9 ± 3.8	53.9 ± 9.0	31.8–70.2	16.7	79

### NGS-Based Whole Genome Resequencing to Generate Sequences for QTL-Seq Study

For QTL-seq study, we performed high-throughput whole genome NGS resequencing of early and late flowering parents as well as bulks- EDTFB (mean DTF: 22.7–28.6 days) and LDTFB (63.0–95.1 days)- of two RIL mapping populations [(ICC 4958 × ICC 17163) and (ICC 4958 × ICC 8261)]. Considering the significant effect of long- and short-day photoperiod on DTF response in two RIL mapping populations across 2 years at green house, early and late flowering bulks- EDTFB (mean DTF: 25.3–30.1) and LDTFB (69.9–94.6) constituted from two RIL mapping populations were made at these two different photoperiod conditions separately and sequenced at a genome-wide scale for QTL-seq study. This produced 173.8 million high-quality average sequence reads (ranged from 167.4 to 181.4 million reads) with a ∼30-fold sequencing-depth coverage. The sequencing data generated in the present study were submitted to NCBI-sequence read archive (SRA) database^2^ with accession number SRR2229140 for unrestricted public access. About 86.5 to 90.1% sequence reads of these were mapped to unique physical locations of reference *desi* genome with a 73.6% mean coverage (Supplementary Table S1). To reduce the potential bias of read-depth in samples, the uniquely mapped sequence reads obtained from parents and bulks (EDTFB and LDTFB) of two RIL mapping populations were normalized in accordance with read coverage-depth. We measured the overall mapping efficiency of non-redundant uniquely mapped sequence reads individually in mapping parents and bulks based on their sequencing-depth coverage (fold) as well as genome coverage (%) (Supplementary Table S1). This covered ∼22.4-fold mean sequencing depth including 73.6% (544.7 Mb) of *desi* chickpea genome (estimated genome size ∼740 Mb). For QTL-seq analysis, we compared the individual normalized sequence reads generated from mapping parents and bulks (EDTFB and LDTFB) with that of reference *desi* genomic sequences including pseudomolecules to discover homozygous high-quality SNPs.

### Molecular Mapping of QTL-Seq Driven Major DF QTLs in a Mapping Population of ICC 4958 × ICC 17163

**FIGURE 2:**
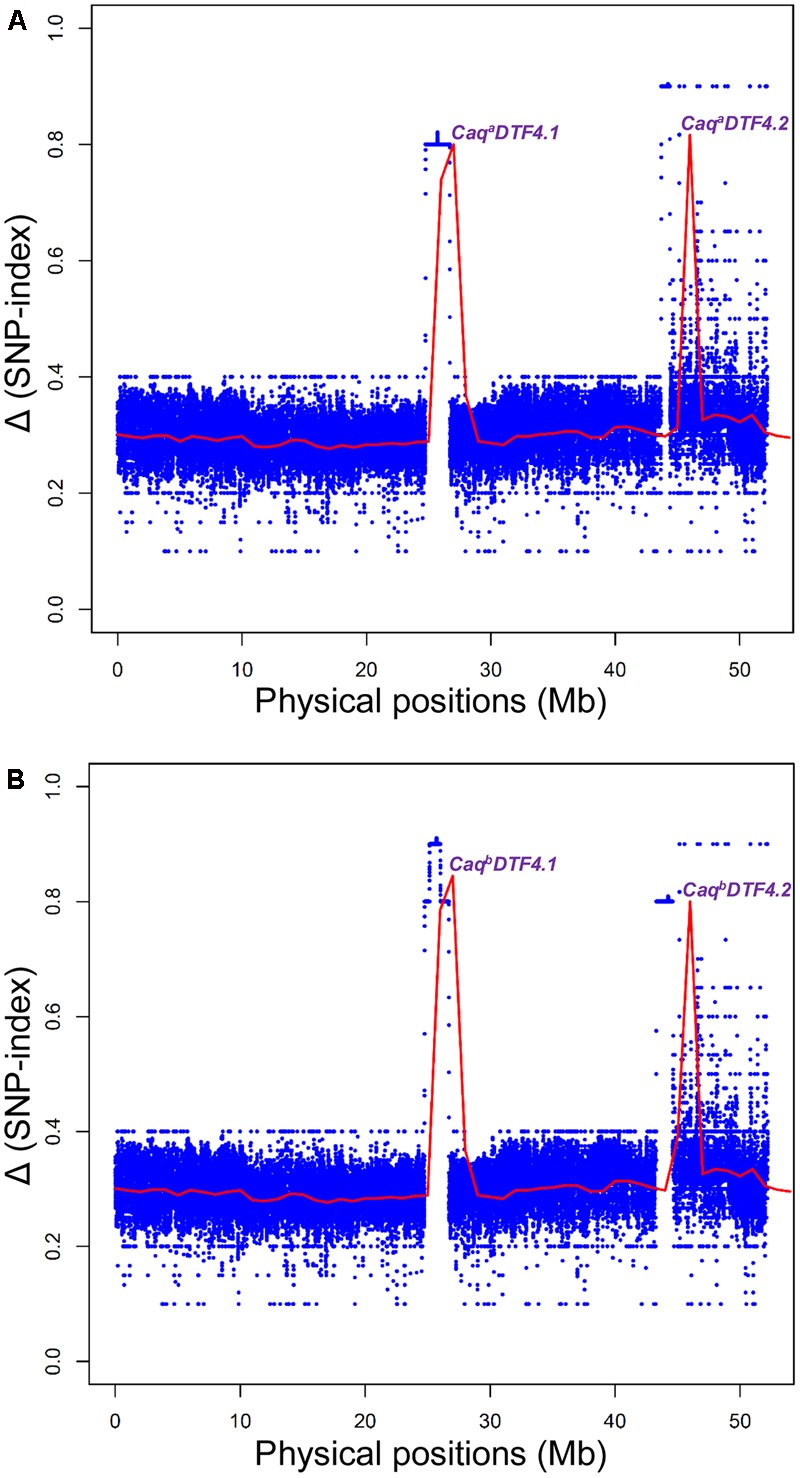
Graphs depicting the Δ (SNP-index) of EDTFB (early days to 50% flowering time bulk) and LDFB (late days to 50% flowering time bulk) obtained from two inter-/intra-specific RIL mapping populations [**A**: (ICC 4958 × ICC 17163) and **B**: (ICC 4958 × ICC 8261)] using multiple QTL-seq assay. The *X*-axis specifies the physical locations (Mb) of *desi* chickpea chromosome 4. The *Y*-axis designates the SNP-index that was measured in accordance with 10 Mb physical interval and 1 kb increment sliding window approach. The null hypothesis-based statistical confidence intervals (significance level at *P* < 0.001) (Takagi et al., 2013; Das et al., 2015a) were used to plot the Δ (SNP-index) which identified two major genomic regions underlying DTF QTLs (*Caq^a^DTF4.1, Caq^a^DTF4.2, Caq^b^DTF4.1*, and *Caq^b^DTF4.2*) from each of the two mapping populations. These major DTF QTLs were defined based on the confidence of significant Δ (SNP-index) > 0.5 (*P* < 0.001) and parameter of SNP-index near to 1 and 0 in EDTFB and LDTFB, respectively.

We discovered 1635117 SNPs (with an average map-density of 0.20 kb) revealing polymorphism between early (ICC 4958 and EDTFB) and late (ICC 17163 and LDTFB) flowering mapping parents and bulks according to their congruent physical positions (bp) on the reference pseudomolecule of *desi* genome (**Table 2** and Supplementary Tables S2, S3). We measured the SNP-index of all individual SNPs exhibiting differentiation between early (ICC 4958 and EDTFB) and late (ICC 17163 and LDTFB) flowering mapping parents and bulks, and plotted these SNP-index against chromosomes of reference genome. A 1-kb sliding window approach was employed to measure the mean SNP-index individually within a 10-Mb target genomic interval. Further, the Δ (SNP-index) was calculated by integrating the SNP-index of EDTFB and LDTFB, which were plotted across the genomic locations (Mb) of reference genome (**Figure 2A**).

**Table 2:** Genomic distribution of SNPs physically mapped on eight chromosomes of *desi* chickpea genome.

		Number (%) of SNPs mapped		Average map density (kb)	
	Size (Mb) of *desi* chromosomes	*Desi* (ICC 4958) vs.	*Desi* (ICC 4958) vs.	*Desi* (ICC 4958) vs.	*Desi* (ICC 4958) vs.
Chromosomes	(pseudomolecules)	Wild (ICC 17163)	*Kabuli* (CDC Frontier)	Wild (ICC 17163)	*Kabuli* (CDC Frontier)
*Ca_Desi_Chr01*	39.9	213381 (13.1)	96877 (16.4)	0.19	0.41
*Ca_Desi_Chr02*	33.2	170028 (10.4)	46869 (7.9)	0.20	0.71
*Ca_Desi_Chr03*	42.3	211478 (12.9)	43852 (7.4)	0.20	0.96
*Ca_Desi_Chr04*	55.0	298242 (18.2)	208734 (35.2)	0.18	0.26
*Ca_Desi_Chr05*	45.8	224449 (13.7)	40685 (6.9)	0.20	1.13
*Ca_Desi_Chr06*	54.8	264837 (16.2)	67588 (11.4)	0.21	0.81
*Ca_Desi_Chr07*	45.3	174065 (10.7)	72792 (12.3)	0.26	0.62
*Ca_Desi_Chr08*	17.7	78637 (4.8)	15089 (2.5)	0.23	1.17
**Total**	**334**	**1635117**	**592486**	**0.20**	**0.56**

**FIGURE 3:**
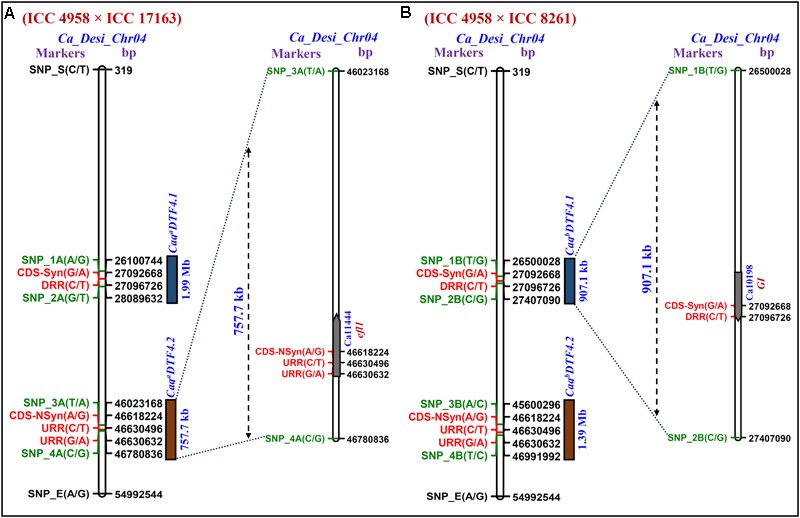
The integration of Δ (SNP-index)-led multiple QTL-seq derived four major flowering time QTLs (*Caq^a^DTF4.1, Caq^a^DTF4.2, Caq^b^DTF4.1*, and *Caq^b^DTF4.2*) in two RIL mapping populations [**A**: (ICC 4958 × ICC 17163) and **B**: (ICC 4958 × ICC 8261)] scaled-down two longer novel major genomic regions underlying two flowering time QTLs, *Caq^a^DTF4.1* and *Caq^b^DTF4.2* into two smaller 757.7 [between flanking SNP markers: SNP_3A (46023168 bp) to SNP_4A (46780835 bp)] and 907.1 [SNP_1B (26500027 bp) to SNP_2B (27407090 bp)] kb sequence intervals (marked by green font), respectively, on *desi* chromosome 4. Consequently, based on highest Δ (SNP-index) value in these multiple QTL-seq derived DTF QTL intervals, three regulatory and synonymous/non-synonymous coding SNP allelic variants-containing two potential *efl1* and *GI* genes (indicated by red font) regulating flowering time were delineated in chickpea.

We identified two major genomic regions (*Caq^a^DTF4.1*: 46023168 to 46780835 bp and *Caq^a^DTF4.2*: 26100745 to 28089632 bp) on chromosome 4 demonstrating the mean SNP-index of ≥ 0.8 in EDTFB and ≤ 0.2 in LDTFB in accordance with the SNP-index measurement criteria defined in QTL-seq analysis (Takagi et al., 2013; Lu et al., 2014; Das et al., 2015a, 2016; **Figures 2A, 3A**). The comprehensive analysis of these selected DTF QTL genomic regions indicated the presence of majority of the SNP alleles derived from early (ICC 4958) and late (ICC 17163) flowering mapping parents in the early and late flowering mapping individuals composing the EDTFB and LDTFB bulks, respectively. Summarily, the QTL-seq assay in an inter-specific mapping population (ICC 4958 × ICC 17163) assured the occurrences of two major DTF QTLs- *Caq^a^DTF4.1* and *Caq^a^DTF4.2*- at the 1.99 Mb [26100745 (SNP_1A) to 28089632 (SNP_2A) bp with a Δ (SNP-index): 0.8] and 757.7 kb [46023168 (SNP_3A) to 46780835 (SNP_4A) bp with a Δ (SNP-index): 0.9] genomic intervals, respectively, on chickpea chromosome 4 (**Figure 3A**).

The detailed structural annotation of 16397 and 2542 SNPs at *Caq^a^DTF4.1* and *Caq^a^DTF4.*2, respectively, revealed the occurrence of 36.4 to 50% of SNPs in the genes and remaining in the intergenic regions (Supplementary Table S4). The gene-derived SNPs comprised of highest and lowest proportion of 72.7–73.5% and 1.1–1.3% SNPs in the DRRs (downstream regulatory regions) and URRs (upstream regulatory regions), respectively. The coding SNPs included the 45.8–54.5% synonymous and 45.5–54.2% non-synonymous (missense and nonsense) SNPs (Supplementary Table S4). The allelic variants of SNPs covering these major DTF QTLs (*Caq^a^DTF4.1* and *Caq^a^DTF4.2*) were further validated by resequencing of PCR fragments amplified from the parents (ICC 4958 and ICC 17163) and mapping individuals forming the EDTFB and LDTFB bulks. Accordingly, two major DTF QTLs were detected on similar aforesaid physical positions of chromosome 4 by the QTL-seq analysis of early- and late-flowering bulks (EDTFB and LDTFB) constituted from a RIL mapping population (ICC 4958 × ICC 17163) using the long- and short-day photoperiod-based greenhouse DTF phenotyping data of chickpea.

### Molecular Mapping of QTL-Seq Driven Major DF QTLs in a Mapping Population of ICC 4958 × ICC 8261

A total of 592486 SNPs (with a mean map-density of 0.56 kb) were found polymorphic between early (ICC 4958 and EDTFB) and late (ICC 8261 and LDTFB) flowering mapping parents and bulks as per their congruent physical locations (bp) on the reference *desi* genome (pseudomolecule) (**Table 2** and Supplementary Tables S2, S3). These genome resequencing-led SNPs were subsequently utilized for QTL-seq analysis.

The SNP-index of individual SNPs exhibiting differentiation between early (ICC 4958 and EDTFB) and late (ICC 8261 and LDTFB) flowering mapping parents and bulks was estimated. The mean SNP-index (within a 1-kb sliding window and 10 Mb genomic interval) as well as Δ (SNP-index) of EDTFB and LDTFB were measured and further plotted across chromosomes as per aforesaid methods (**Figure 2B**). This essentially detected two major genomic regions (*Caq^b^DTF4.1*: 45600294 to 46991993 bp and *Caq^b^DTF4.2*: 26500027 to 27407090 bp) on chromosome 4 revealing the mean SNP-index of ≥ 0.8 in EDTFB and ≤ 0.2 in LDTFB (**Figures 2B, 3C,D**). The accuracy of these major genomic regions underlying DTF QTLs was ascertained by a valid 99% Δ (SNP-index) significance level. The comprehensive analysis of these DTF QTL genomic regions inferred the occurrence of majority of the SNP alleles derived from parents (ICC 4958 and ICC 8261) in early and late flowering mapping individuals forming the EDTFB and LDTFB bulks, respectively. Overall, the QTL-seq in an intra-specific mapping population (ICC 4958 × ICC 8261) identified two major DTF QTLs- *Caq^b^DTF4.1* and *Caq^b^DTF4.2*- at the 907.1 kb [26500027 (SNP_1B) to 27407090 (SNP_2B) bp with a Δ (SNP-index): 0.9] and 1.39 Mb [45600294 (SNP_3B) to 46991993 (SNP_4B) bp with a Δ (SNP-index): 0.8] genomic intervals, respectively, on chickpea chromosome 4 (**Figure 3B**).

The detailed structural annotation of 7302 and 3177 SNPs at *Caq^b^DTF4.1* and *Caq^b^DTF4.2*, respectively, revealed the occurrence of 32.5 to 49.1% of SNPs in the genes and rest in the intergenic regions (Supplementary Table S4). The gene-derived SNPs included highest and lowest proportion of 73.8–74.6% and 1.2–1.5% SNPs in the DRRs and URRs, respectively. The coding SNPs included the 52.1–54.7% synonymous and 45.3–47.9% non-synonymous (missense and nonsense) SNPs (Supplementary Table S4). The allelic variants of SNPs covering the QTL-seq led major DTF QTLs (*Caq^b^DF4.1* and *Caq^b^DF4.2*) were validated by resequencing of PCR fragments amplified from the parents (ICC 4958 and ICC 8261) and mapping individuals composing the EDTFB and LDTFB bulks. Like-wise, we detected two major DTF QTLs on similar aforementioned physical positions of chromosome 4 by the QTL-seq analysis of EDTFB and LDTFB bulks constituted from a RIL mapping population (ICC 4958 × ICC 8261) using the long- and short-day photoperiod-based greenhouse DTF phenotyping data of chickpea.

### Multiple QTL-Seq Rapidly Delineates Candidate Genes and Natural Allelic Variants Regulating Flowering Time in Chickpea

We correlated and compared the four major genomic regions underlying DTF QTLs (*Caq^a^DTF4.1, Caq^a^DTF4.2, Caq^b^DTF4.1* and *Caq^b^DTF4.2*) identified and mapped by QTL-seq in two intra-/inter-specific RIL mapping populations of ICC 4958 × ICC 17163 and ICC 4958 × ICC 8261. Based on these analyses, two consensus major short physical genomic intervals of 757.7 kb [46023168 (SNP_3A) to 46780835 (SNP_4A) bp] and 907.1 kb [26500027 (SNP_1B) to 27407090 (SNP_2B) bp] harboring the major DTF QTLs were detected on chromosome 4 (**Figures 3A,B**). Our comprehensive multiple QTL-seq analysis in two inter-/intra-specific RIL mapping populations ascertained the validity of novel natural allelic variants-containing similar *efl1* and *GI* genes with a highest Δ (SNP-index) of 1.0 at *Caq^a^DTF4.2* and *Caq^b^DTF4.1* QTL regions governing flowering time in chickpea. Henceforth, these strong flowering time-associated *efl1* and *GI* genes localized at a major DTF QTL interval (*Caq^a^DTF4.2* and *Caq^b^DTF4.1*) were considered as the potential candidates for flowering time regulation in chickpea. This essentially identified two upstream regulatory [46630632 (G/A) and 46630495 (C/T) bp] and one non-synonymous [Asparagine (AAT) to Serine (AGT)] coding [46618224 (A/G) bp] SNP allelic variants from a *efl1 desi* gene (Ca11444) as well as two synonymous coding [27092669 (G/A) bp] and downstream regulatory [27096726 (C/T) bp] SNP alleles from a *GI desi* gene (Ca10198) regulating flowering time in chickpea (**Figures 3A,B** and Supplementary Figure S1).

### Natural Allelic Diversity-Led Domestication Pattern in Flowering Time Genes

The novel SNP allelic variants discovered from the coding (synonymous and non-synonymous) and non-coding regulatory sequence regions of two flowering genes, *efl1* (117 SNPs) and *GI* (31) localized at two major DTF QTL regions (identified by multiple QTL-seq) were genotyped in 93 *desi* and *kabuli* cultivated and 79 wild chickpea accessions to determine their natural/functional allelic diversity-based domestication pattern based on multiple nucleotide diversity parameters (𝜃π and Tajima’s D) (Supplementary Table S5). The coding and regulatory SNPs discovered from the *efl1* (36–49% phenotypic variation explained) and *GI* (33–42%) flowering genes exhibited significantly higher association potential for early and late DTF differentiation among chickpea accessions (**Table 3**). Notably, only 9 to 12% of natural allelic variation-based functional diversity level estimated in *efl1* and *GI* flowering genes among wild gene pool was retained and thus got preserved in cultivated chickpea. The relative mean natural allelic diversity of two flowering genes (*efl1* and *GI*) localized at major DTF QTL regions between cultivated and wild chickpea varied from 88 to 91% (𝜃πCc/𝜃πCw). This was much higher than the relative mean natural allelic diversity level (𝜃πCc/𝜃πCw: 61.8%) estimated by using 7116 genome-wide SNPs localized at genomic regions where no DTF QTLs were mapped.

**Table 3:** Gene-derived SNP alleles associated with days to 50% flowering time (DTF) detected by association mapping in chickpea.

							Association analysis		
	*Kabuli*	SNP physical		Gene	Structural	Encoded gene		PVE	Traits
SNP IDs^∗^	chromosomes	positions (bp)	SNPs	accession IDs	annotation	(protein)	*P*	(%)	associated
Ca_LG_446618224	*Ca_Kabuli_Chr4*	46618224	A	Ca11444	CDS (Non- synonymous)	*efl1* (early flowering 1)	1.56 × 10^-13^	36	EDTF
		46618224	G				1.43 × 10^-12^	38	LDTF
Ca_LG_446630495	*Ca_Kabuli_Chr4*	46630495	C	Ca11444	URR	*efl1* (early flowering 1)	1.07 × 10^-12^	49	EDTF
		46630495	T				1.13 × 10^-10^	47	LDTF
Ca_LG_446630632	*Ca_Kabuli_Chr4*	46630632	G	Ca11444	URR	*efl1* (early flowering 1)	1.24 × 10^-12^	41	EDTF
		46630632	A				1.37 × 10^-12^	40	LDTF
Ca_LG_427092669	*Ca_Kabuli_Chr4*	27092669	G	Ca10198	CDS (Synonymous)	*GI* (GIGANTEA)	1.17 × 10^-10^	35	EDTF
		27092669	A				1.22 × 10^-10^	33	LDTF
Ca_LG_427096726	*Ca_Kabuli_Chr4*	27096726	C	Ca10198	DRR	*GI* (GIGANTEA)	1.30 × 10^-11^	43	EDTF
		27096726	T				1.40 × 10^-10^	42	LDTF

*^∗^Correspond to SNP IDs mentioned in Supplementary Tables S2, S3. EDTF/LDTF, early/late DTF. PVE, phenotypic variation explained.*

## Discussion

A broader phenotypic variation coupled with bi-directional transgressive segregation (normal frequency distribution) of DTF trait among RIL individuals and parents of inter (ICC 4958 × ICC 17163)- and intra (ICC 4958 × ICC 8261)-specific mapping population phenotyped in field and green house (long- and short-day) conditions at two different geographical locations/years was evident. This infers the complex genetic inheritance pattern of flowering time quantitative trait in chickpea. Therefore, genetic dissection of this complex quantitative trait employing various genomics-assisted breeding strategies is essential for genetic enhancement and to develop early flowering high seed and pod-yielding stress tolerant cultivars of chickpea during present scenario of climate change. To accomplish these, our study selectively employed a rapid, cost-efficient and NGS-led high-throughput multiple QTL-seq assay in two inter- and intra-specific RIL mapping population exhibiting a much wider flowering time trait variation including a higher heritability (consistent phenotypic expression) for flowering time in field and green house (long- and short-day) across two diverse geographical locations/years in order to identify major flowering time QTLs in chickpea.

The QTL-seq analysis in an inter-specific RIL mapping population (ICC 4958 × ICC 17163) detected 1.99 Mb and 757.7 kb two major genomic regions underlying *Caq^a^DTF4.1* and *Caq^a^DTF4.2* QTLs, respectively, mapped on chromosome 4 governing flowering time in chickpea. Like-wise, 907.1 kb and 1.39 Mb, two major genomic intervals of *Caq^b^DTF4.1* and *Caq^b^DTF4.2* QTLs, respectively, mapped on chromosome 4 were detected by QTL-seq analysis in an intra-specific RIL mapping population (ICC 4958 × ICC 8261). These analyses altogether led to identify two consensus major short physical genomic regions of 757.7 kb and 907.1 kb harboring *Caq^a^DTF4.2* and *Caq^b^DTF4.1* QTLs, respectively, on chromosome 4 of chickpea. The validation of these major DTF QTLs across two diverse intra-/inter-specific chickpea mapping population was apparent implicating the robustness of identified QTLs in regulating flowering time in chickpea. The aforesaid outcomes also inferred the efficacy of multiple QTL-seq assay to narrow-down the longer major DTF QTL intervals detected by QTL-seq in an individual mapping population into shorter major QTL regions in multiple mapping populations of chickpea. This suggests the potential utility of multiple QTL-seq over NGS-based QTL-seq assay and other conventional QTL mapping approaches in high-resolution molecular/fine mapping of major genomic regions harboring QTLs governing diverse agronomic traits including flowering time in chickpea. As per congruent physical positions (bp) on *desi* chromosome 4, two short interval DTF QTLs (*Caq^a^DTF4.2* and *Caq^b^DTF4.1*) revealed correspondence with the two earlier identified known major flowering time QTLs (*CaqDF4.1* and *CaqDF4.2*) that are identified and mapped on an intra-specific high-density genetic linkage map (ICC 16374 × ICC 762) of chickpea (Upadhyaya et al., 2015).

The comprehensive multiple QTL-seq analysis at *Caq^a^DTF4.2* and *Caq^b^DTF4.1* QTL regions detected novel natural allelic variants-containing two strong flowering time-associated *efl1* and *GI* genes with highest Δ (SNP-index) of 1.0 and thereby, considered as the potential candidates for flowering time regulation in chickpea. Two potential candidate genes, *efl1* and *GI* underlying these major QTLs (*Caq^a^DTF4.2/CaqDF4.1* and *Caq^b^DTF4.1*/*CaqDF4.2* detected in our present and past studies, respectively) regulating flowering time have been delineated by deploying an integrated genomics-assisted breeding strategy involving candidate gene-based trait association mapping, GWAS, QTL mapping, differential transcript expression profiling and gene-specific molecular haplotyping in chickpea (Upadhyaya et al., 2015). The potential of these identified known flowering development pathway and *FT* gene homologs like *efl1* and *GI* colocalized at the major QTLs in regulating flowering time have been documented by different traditional QTL mapping studies involving diverse intra- and inter-specific mapping populations of chickpea (Cho et al., 2002; Anbessa et al., 2006; Lichtenzveig et al., 2006; Cobos et al., 2007, 2009; Radhika et al., 2007; Aryamanesh et al., 2010; Hossain et al., 2010; Gowda et al., 2011; Rehman et al., 2011; Hiremath et al., 2012; Vadez et al., 2012; Jamalabadi et al., 2013; Zhang et al., 2013; Varshney et al., 2014). Notably, functional validation and comprehensive molecular characterization of photoperiod-independent *efl1* gene and photoperiod-dependent circadian-clock-related *GI* gene have implicated their potential involvement in regulating flowering time of legumes and *Arabidopsis* (Hecht et al., 2007; Liu et al., 2008; Watanabe et al., 2009, 2011, 2012; Weller et al., 2009; Kang et al., 2010; Laurie et al., 2011; Andres and Coupland, 2012; Kim et al., 2012; Pin and Nilsson, 2012; Song et al., 2013; Yamashino et al., 2013; Zhai et al., 2014; Weller and Martínez, 2015).

Despite of identifying similar flowering time *efl1* and *GI* genes between past and present studies, we were able to discover diverse novel flowering time-regulating non-synonymous and regulatory natural SNP allelic variants (unlike our previous study by Upadhyaya et al., 2015) from the two target *efl1* and *GI* genes that are localized in the two multiple QTL-seq derived major DTF QTL regions (*Caq^a^DTF4.2* and *Caq^b^DTF4.1*) of chickpea. The detection of altogether different natural allelic variants from two similar *efl1* and *GI* flowering genes localized at two major DTF QTL regions between past and present studies collectively infers the population/cultivar-specific genetic inheritance pattern of complex flowering time quantitative trait in diverse genetic backgrounds of chickpea. The above clues collectively suggest the accuracy, robustness and wider practical applicability of multiple QTL-seq approach for fast genome-wide scanning and mapping of high-resolution major flowering time QTLs as well as delineation of candidate genes and novel natural alleles underlying these major QTLs governing flowering time in chickpea.

The quantitative flowering time trait is primarily governed by complex regulatory networks/pathways involving a diverse array of genes in plant species including legumes (Andres and Coupland, 2012; Song et al., 2013; Weller and Martínez, 2015). The molecular haplotyping of *efl1* and *GI* genes (detected by multiple QTL-seq) among diverse *desi* and *kabuli* cultivated and wild accessions has detected multiple novel natural allelic variants including haplotypes in these flowering time genes exhibiting varied potential characteristics for flowering time trait regulation and evolutionary pattern in domesticated chickpea (Upadhyaya et al., 2015). Therefore, novel functionally relevant potential molecular signatures (SNP markers, genes, QTLs and natural allelic variants) governing flowering time delineated by us employing a NGS-based high-throughput multiple QTL-seq strategy can be useful for fast genetic dissection of complex flowering time quantitative trait and eventually genomics-assisted crop improvement to develop early flowering varieties of chickpea with limited resource expenses.

Preliminary efforts have been made to understand the natural/functional allelic diversity-based domestication pattern of two flowering (*efl1* and *GI*) genes localized at two major DTF QTL regions among *Cicer* cultivated (*desi* and *kabuli*) and wild genepools. This exhibited a significant reduction of natural/functional allelic diversity in cultivated *desi* and *kabuli* accessions from diverse geographical regions of the world as compared to annual and perennial wild accessions belonging to primary, secondary and tertiary gene pools of chickpea. However, this observed background allelic diversity reduction in cultivated than that of wild chickpea was much stronger especially at major DTF QTL regions where *efl1* and *GI* flowering genes were mapped. This implicates that these genes were targeted by artificial selection which was further evident from their non-neutral evolution during chickpea domestication based on significant variation of Tajima’s D between cultivated (–1.12) and wild (0.26) accessions, respectively. Interestingly, all these natural allelic variants-containing two potential genes localized within flowering time major QTL intervals have been commonly identified and mapped on multiple independent chickpea mapping populations by earlier and our present studies (Upadhyaya et al., 2015). These outcomes clearly reflect the extensive contribution of four sequential evolutionary bottlenecks including vernalization and strong artificial and/or natural selection pressure on these flowering time-associated natural allelic variants of two gene loci (*efl1* and *GI*) during chickpea domestication leading toward reduction of genetic diversity in cultivated chickpea as compared to that of wild *Cicer* genepool (Lev-Yadun et al., 2000; Abbo et al., 2003; Berger et al., 2005; Burger et al., 2008; Toker, 2009; Meyer et al., 2012; Jain et al., 2013; Kujur et al., 2013; Varshney et al., 2013; Saxena et al., 2014a,b).

The most crucial evolutionary bottleneck, vernalization, is a vital key module of flowering time during chickpea domestication culminating into existence of currently cultivated vernalization insensitive *desi* and *kabuli* cultivars specifically from the vernalization sensitive wild ancestor *C. reticulatum* (Abbo et al., 2003, 2014; Berger et al., 2005; Burger et al., 2008; Toker, 2009). The major domestication bottlenecks integrated with artificial selection including modern breeding efforts have been constantly practiced during the chickpea genetic improvement program for developing its early flowering cultivars of high seed and pod yield. These findings collectively infer that the natural allelic variation-based functional diversity scanned in the genes might be associated with flowering time trait evolution with regard to differential domestication-led bottlenecks including vernalization response in *desi* and *kabuli* cultivated and wild chickpea during domestication. Henceforth, flowering time represents a vital component of domestication trait selected during breeding and genetics of chickpea. Moreover, the major impact of long- and short-photoperiods which are the major environmental cues for determining the flowering time including time of flower initiation and/or first flower appearance especially in photoperiod-sensitive as compared to photoperiod-insensitive chickpea accessions is well documented (Daba et al., 2016). In the present study, a significant interactions between long- and short-day photoperiods and DTF trait variation observed in individuals of two RIL mapping populations across 2 years was apparent. In spite of this concern, we were able to identify functionally relevant non-synonymous/synonymous coding and regulatory SNP allelic variants from two flowering genes (*efl1* and *GI*) localized at two major DTF QTL regions by using the long- and short-day phenotyping data of both RIL populations separately in multiple QTL-seq assay. This further infers the efficacy of strategy (multiple QTL-seq) implemented in our study to detect potential molecular signatures regulating flowering time in chickpea. It is, therefore, essential to perform a comprehensive analysis using all natural/functional allelic variants discovered and potential locus targeted by natural and/or artificial selection in two flowering genes (*efl1* and *GI*) to delve deeper into the complex flowering time trait evolution and domestication in chickpea. This will be useful to understand the molecular mechanism influencing fixation of such complex flowering time quantitative trait in domesticated cultivars that are adapted to multiple agro-ecological regions of the world and further pave the way for genetic enhancement to develop early flowering high seed/pod-yielding varieties of chickpea amidst current climate change scenario.

## Author Contributions

RS, AD, and UB conducted all experiments and drafted the manuscript. HU, RK, PS, ST, and CB helped in development, advancement and phenotyping of mapping populations. AT and SP conceived and designed the study, guided data analysis and interpretation, participated in drafting and correcting the manuscript critically and gave the final approval of the version to be published. All authors have read and approved the final manuscript.

## Conflict of Interest Statement

The authors declare that the research was conducted in the absence of any commercial or financial relationships that could be construed as a potential conflict of interest.

## Footnotes

1 http://www.sequenom.com

2 http://www.ncbi.nlm.nih.gov/sra

## Supplementary Material

The Supplementary Material for this article can be found online at: http://journal.frontiersin.org/article/10.3389/fpls.2017.01105/full#supplementary-material. For Tables S2 and S3, see: https://figshare.com/s/f1848022c4f3c620f1d2.
