# Supplementary material for: A Multiple QTL-Seq Strategy Delineates Potential Genomic Loci Governing Flowering Time in Chickpea
Source: Front Plant Sci. 2017 Jul 11;8:1105. doi: 10.3389/fpls.2017.01105 (PMC5508101; doi:10.3389/fpls.2017.01105)
Supplement: Supplementary file 1 [file Data_Sheet_1.zip › Table 1.PDF]

Table S1. Summary of sequencing statistics generated by resequencing of early and late flowering parental accessions and bulks from two mapping populations

**Characteristics**	**ICC 4958 × ICC 17163**				**ICC 4958 × ICC 8261**				**Mean**
	**ICC 4958**	**ICC 17163**	**EDFTB**	**LDFTB**	**ICC 4958**	**ICC 8261**	**EDFTB**	**LDFTB**	
Millions (Mb) of high-quality sequence-reads generated	181.4 (23763)	178.5 (23026)	171.9 (21487)	167.4 (21260)	181.4 (23763)	180.5 (22811)	170.6 (20054)	177.2 (21441)	175.4 (22201)
Sequencing depth of coverage (fold) obtained by high-quality sequence-reads	32.1	31.1	29.0	28.7	32.1	30.8	27.1	28.9	30.0
Number (%) of high-quality sequence-reads mapped onto reference *desi* chickpea genome	173.3 (95.5)	167.5 (93.8)	164.6 (95.7)	157.3 (94.0)	173.3 (95.5)	171.6 (95.1)	161.6 (94.7)	167.7 (94.6)	166.2 (94.8)
Size (Mb) of high-quality sequence-reads mapped onto reference *desi* chickpea genome	20509.7	19043.9	21221.9	22626.8	20509.7	19858.8	18451.8	19490.1	20214.1
Number (%) of high-quality sequence-reads uniquely mapped onto reference *desi* chickpea genome	136.3 (75.1)	131.5 (73.7)	130.6 (75.9)	125.3 (74.8)	136.3 (75.1)	130.6 (72.4)	120.6 (70.7)	128.7 (72.6)	129.1 (73.6)
Genome coverage (Mb) by uniquely mapped sequence reads	17462.7	17001.9	16182.9	16589.8	17462.7	16817.8	15400.8	16441.1	16556.7
Genomic regions (Mb) [%] covered (considering estimated chickpea genome size of ~ 740 Mb)	555.9 [75.1]	545.2 [73.7]	562.0 [75.9]	553.9 [74.8]	555.9 [75.1]	535.5 [72.4]	523.1 [70.7]	537.5 [72.6]	544.7 [73.6]
Sequencing depth of coverage (fold) of whole *desi* chickpea genome	23.6	23.0	21.9	22.4	23.6	22.7	20.8	22.2	22.4

EDTFB/LDTFB: Early/Late days to 50% flowering time bulk
