# Supplementary material for: A Multiple QTL-Seq Strategy Delineates Potential Genomic Loci Governing Flowering Time in Chickpea
Source: Front Plant Sci. 2017 Jul 11;8:1105. doi: 10.3389/fpls.2017.01105 (PMC5508101; doi:10.3389/fpls.2017.01105)
Supplement: Supplementary file 1 [file Data_Sheet_1.zip › Table 4.PDF]

Table S4. SNPs annotated in diverse coding and non-coding sequence components of genes as well as intergenic regions of major DTF QTL genomic intervals detected using multiple QTL-seq strategy

**Major DTF QTLs identified through multiple QTL-seq assay**	**Total number of SNPs detected**	**Intergenic-SNPs [%]**	**Genic-SNPs (Genes) [%]**	**URR-SNPs (Genes) [%]**	**Intron-SNPs (Genes) [%]**	**DRR-SNPs (Genes) [%]**	**CDS-SNPs (Genes) [%]**	**Synonymous-SNPs (Genes) [%]**	**Non-synonymous-SNPs (Genes) [%]**
Caq[a]*DTF4.1*	16397	8200 [50]	8197 (166) [50]	87 (42) [1.1]	1568 (96) [19.1]	5963 (165) [72.7]	579 (91) [7.1]	265 (74) [45.8]	314 (72) [54.2]
Caq[a]*DTF4.2*	2542	1617 [63.6]	925 (33) [36.4]	12 (6) [1.3]	167 (13) [18.1]	680 (32) [73.5]	66 (15) [7.1]	36 (14) [54.5]	30 (9) [45.5]
Caq[b]*DTF4.1*	7302	3716 [50.9]	3586 (74) [49.1]	53 (22) [1.5]	663 (45) [18.5]	2676 (74) [74.6]	194 (41) [5.4]	101 (32) [52.1]	93 (30) [47.9]
Caq[b]*DTF4.2*	3177	2143 [67.5]	1034 (56) [32.5]	12 (6) [1.2]	184 (21) [17.8]	763 (53) [73.8]	75 (20) [7.2]	41 (17) [54.7]	34 (12) [45.3]
