# Supplementary material for: A Multiple QTL-Seq Strategy Delineates Potential Genomic Loci Governing Flowering Time in Chickpea
Source: Front Plant Sci. 2017 Jul 11;8:1105. doi: 10.3389/fpls.2017.01105 (PMC5508101; doi:10.3389/fpls.2017.01105)
Supplement: Supplementary file 1 [file Data_Sheet_1.zip › Table 5.PDF]

Table S5. SNPs mined from diverse coding and non-coding sequence components of two flowering time-associated genes localized at major DTF QTL genomic intervals detected by multiple QTL-seq strategy

**Flowering time-associated genes**	**Gene Accession IDs**	**Total number of SNPs detected**	**URR-SNPs (%)**	**Intron-SNPs (%)**	**DRR-SNPs (%)**	**CDS-SNPs (%)**	**Synonymous-SNPs (%)**	**Non-synonymous SNPs (%)**
efl1 (early flowering 1)	Ca11444	117	3 (2.6)	62 (53.0)	34 (29.0)	18 (15.4)	13 (72.2)	5 (27.8)
GI (GIGANTEA)	Ca10198	31	0	10 (32.3)	19 (61.3)	2 (6.4)	2 (100)	0
