# Supplementary material for: A Multiple QTL-Seq Strategy Delineates Potential Genomic Loci Governing Flowering Time in Chickpea
Source: Front Plant Sci. 2017 Jul 11;8:1105. doi: 10.3389/fpls.2017.01105 (PMC5508101; doi:10.3389/fpls.2017.01105)
Supplement: Supplementary file 1 [file Data_Sheet_1.zip › Image 1.PDF]

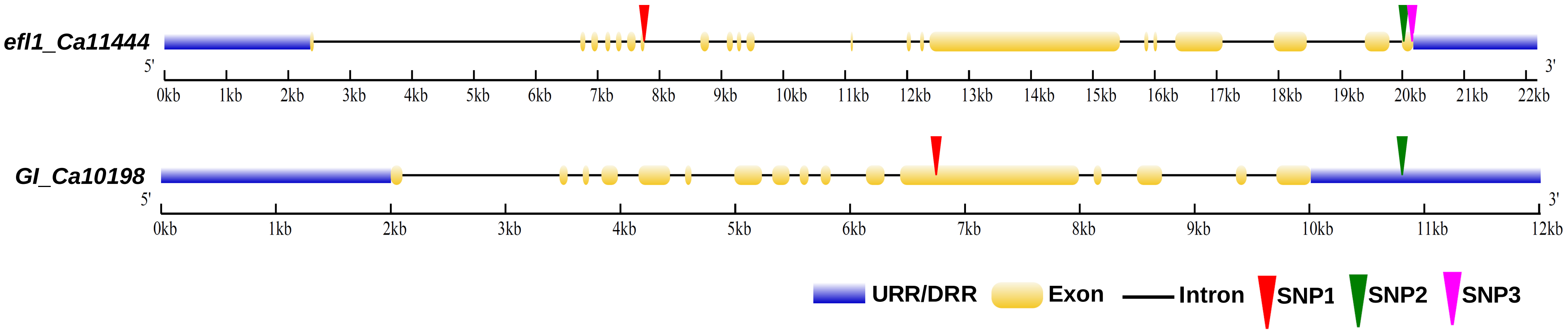

Figure S1. Detail structural annotation of SNP allelic variants-containing two *efl1* (early flowering 1) and *GI* (GIGANTEA) genes regulating days to 50% flowering time (DTF) delineated at multiple QTL-seq derived two major DTF QTL regions, *Caq*^{a}*DTF4*.2 and *Caq*^{b}*DTF4.1*, respectively in chickpea.
